# Transformational Leadership, Psychological Empowerment, and Organizational Citizenship Behaviors among Nursing Workforce: A Single Mediation Analysis

**DOI:** 10.1155/2024/9919371

**Published:** 2024-05-03

**Authors:** Ibrahim Abdullatif Ibrahim, Ahmed Hashem El-Monshed, Marwan Altheeb, Mohamed Gamal El-Sehrawy

**Affiliations:** ^1^Nursing Department, College of Applied Medical Sciences, Shaqra University, Shaqra, Saudi Arabia; ^2^Department of Nursing Administration, Faculty of Nursing, Mansoura University, Mansoura, Egypt; ^3^Department of Nursing, College of Health and Sport Sciences, University of Bahrain, Manama, Bahrain; ^4^Department of Psychiatric and Mental Health Nursing, Faculty of Nursing, Mansoura University, Mansoura, Egypt; ^5^Department of Nursing, College of Applied Medical Sciences, Prince Sattam Bin Abdulaziz University, AlKharj, Saudi Arabia; ^6^Department of Nursing Administration, Faculty of Nursing, Port Said University, Port Said, Egypt

## Abstract

**Aim:**

To explore the mediating effect of psychological empowerment in the association between transformational leadership and organizational citizenship behaviors in nursing context.

**Background:**

Healthcare organizations worldwide are facing unprecedented challenges, necessitating effective leadership strategies to ensure quality patient care and organizational success. Transformational leadership has emerged as a tool to promote positive workplace behaviors, including organizational citizenship behaviors, among nursing staff. However, the mediating role of psychological empowerment in this relationship remains underexplored.

**Methods:**

A cross-sectional survey was conducted from March 2023 until August 2023 involving 305 registered nurses at King Khalid Hospital to investigate the relationship among transformational leadership, psychological empowerment, and organizational citizenship behaviors. Validated scales were used to assess these variables. A single mediation analysis was conducted through processing macro version 3.5 model 4.

**Results:**

This study found a strong positive association between transformational leadership and both psychological empowerment (*r* = 0.507, *p* < 0.001) and organizational citizenship behaviors (*r* = 0.445, *p* < 0.001) among nursing staff. Additionally, psychological empowerment partially mediated the relationship between transformational leadership and organizational citizenship behaviors, with a significant indirect effect (*B* = 0.110, CI: 0.058–0.166).

**Conclusions:**

Transformational leadership positively impacted nurses' feelings of empowerment, which then led to higher exhibition of organizational citizenship behaviors. *Implications for Nursing Management*. Leadership development programs should prioritize the cultivation of transformational leadership qualities and support the psychological empowerment of nursing staff. This approach can enhance organizational effectiveness, foster positive workplace environments, and improve patient outcomes.

## 1. Introduction

Healthcare is currently experiencing substantial transformation due to the prevailing economic challenges worldwide. At the same time, demographic patterns are evolving, with a notable increase in the aging population in the most affluent nations of the industrialized world, combined with a growing global population. In this dynamic healthcare landscape, the ability to meet rigorous standards is paramount and this necessitates the implementation of effective leadership styles [1, 2].

Nurse leadership emerges as a particularly crucial element in this unpredictable and occasionally tumultuous world of healthcare. Nurse leaders play a vital role in ensuring the delivery of safe, evidence-based treatment that enhances the overall patient experience. Their guidance and expertise contribute to maintaining high standards of care and promoting positive patient outcomes [3]. Throughout history, nurse leaders have consistently demonstrated strong management abilities. However, to truly excel as a leader, one must possess transformational leadership skills. This entails the ability to inspire and motivate individuals on a personal level, encouraging them to take innovative actions that lead to the attainment of optimal outcomes. By embodying transformational leadership qualities, nurse leaders can foster a culture of innovation, collaboration, and continuous improvement, ultimately enhancing the quality of care and patient outcomes [4].

Transformational leaders exhibit effective communication, inspire others, demonstrate enthusiasm, foster positive change, and guide individuals towards shared objectives that enhance nurses' wellbeing [5]. Transformational leadership is crucial for achieving exceptional organizational performance and efficiency. Additionally, transformational leaders can significantly improve staff satisfaction by providing support and motivation, empowering employees, encouraging constructive feedback, fostering open communication, and demonstrating respect [6]. Transformational leadership inspires people to surpass their usual duties, promoting flexibility and dedication to a shared goal or mission. These additional efforts, referred to as organizational citizenship behaviors (OCBs), help strengthen the workplace environment by exceeding expectations and have been associated with enhanced organizational performance [7].

Moreover, transformational leaders recognize the importance of empowering their staff by delegating authority, fostering accountability, and involving employees in decision-making processes. This acknowledges the increasing need to promote OCBs to maximize the efficient use of limited resources [7, 8].

Concurrently, OCBs have been observed as a significant predictor of employees' productive work [9]. To foster these kinds of behaviors, organizations must provide additional consideration. For instance, when employees lack sufficient information and control over their job duties, have limited involvement in decision-making, experience communication issues, or feel dissatisfied with leadership, it can exacerbate negative behaviors such as turnover. These factors directly oppose the principles of psychological empowerment [10].

In this study, we aim to investigate the influence of transformational leadership on OCBs through psychological empowerment. Specifically, we argue that transformational leaders can enhance nurses' psychological empowerment by elevating the significance they attribute to their work, equipping them with the necessary skills for task completion, and fostering a sense of control over their environment. This assumption builds on the theoretical idea of social exchange theory [11]. According to social exchange theory, employees engage in discretionary behaviors, such as OCBs, when they perceive that their efforts are valued and rewarded by their leaders or the organization. Transformational leadership, which emphasizes inspiring and empowering followers, can foster a supportive work environment where employees feel psychologically empowered to engage in OCBs as a form of reciprocal exchange for the positive leadership they receive.

Previous studies have investigated the distinct associations among transformational leadership, psychological empowerment, and OCBs [12, 13]. Nevertheless, there exists a lack of research concerning the manner in which psychological empowerment serves as a mediator in the relationship between transformational leadership and OCBs, particularly within the nursing workforce. This study will contribute to the existing literature by providing insights into the mediating role of psychological empowerment in the relationship between transformational leadership and OCBs within the nursing context. By examining the mediating pathways, this research will enhance our understanding of the mechanisms underlying these relationships, offering valuable implications for healthcare management and leadership development.

### 1.1. Literature Review and Hypotheses Development

#### 1.1.1. Transformational Leadership and OCBs

The hallmark of a transformational leader is their capacity to uplift and encourage subordinates to reach greater heights of achievement and personal development [5]. A multitude of studies have been conducted on the transformational leadership style, consistently demonstrating its significant impact on employee commitment, satisfaction, and overall organizational performance. The findings consistently highlight the positive association between transformational leadership and employee outcomes, such as increased job satisfaction, higher levels of organizational commitment, and improved performance [14, 15].

A prior research has demonstrated that positive outcomes in nursing organizations depend on the adoption of a transformational leadership approach. This style effectively leads nursing staff, addresses staffing concerns, and improves nurse retention [16]. The encouragement and promotion of reasoned thought as a leadership quality have a significant impact on employees' creative behavior and foster collaboration [17]. Empirical data derived from a study encompassing a total of 219 nurses demonstrate that transformational leadership possesses a significant impact on the advancement of OCBs within the nursing profession [18]. Correspondingly, Zurahmi et al. [19] emphasize the intricate connection between transformational leadership and the occurrence of OCBs among nursing staff at the Tapan Regional General Hospital. Moreover, transformational leadership plays a substantial role in fostering the emergence of employee OCBs [20]. As a result, we have formulated the following hypothesis:

  H1: Transformational leadership will have a positive effect on OCBs among nurses

#### 1.1.2. Transformational Leadership and Psychological Empowerment

Transformational leaders in nursing play a vital role in supporting change processes that advance various goals, including enabling high-quality care, ensuring patient safety, and improving the overall quality of life for nurses in the workplace. To achieve these outcomes, managers must provide nurses with the autonomy and freedom to perform their duties in alignment with best practices. This involves empowering nurses to make decisions, take ownership of their work, and contribute to the organization's mission. Psychological empowerment for nurses is not only necessary but also has a significant influence on work performance. When nurses feel empowered, they are more likely to be engaged, motivated, and committed to delivering exceptional care, thereby positively impacting their overall work performance [21]. Given its profound positive effects on both organizations and their personnel, psychological empowerment has emerged as a compelling subject for practical application and scholarly inquiry, driving further exploration and examination in the field [22]. In 1995, Spreitzer was proposed four components to conceptualize psychological empowerment, namely, meaning, competence, self-determination, and impact. Additionally, a resilient and dependable survey was formulated to expedite the economic evaluation of psychological empowerment [23].

The study conducted by Aydogmus et al. [24] sheds light on the relationship between transformational leadership and psychological empowerment, highlighting the significant influence leaders have on employees' perceptions and experiences. Additionally, the research conducted by Cheng et al. [25] further emphasizes the role of transformational leadership as an influential factor in shaping employees' psychological empowerment. These findings contribute to our understanding of how transformational leadership behaviors can foster a sense of empowerment among employees, ultimately influencing their attitudes, behaviors, and overall well-being in the workplace.

Transformational leaders have the remarkable ability to instill faith in their team members' abilities to achieve objectives, thereby strengthening followers' sense of competence. This sense of competence fosters a strong personal dedication towards their objectives and propels individuals with an inner drive, making them less reliant on external oversight to carry out their duties. As a result, the accomplishment of individual goals amplifies one's impact and enhances their sense of autonomy [22]. Furthermore, as transformational leaders, they empower their followers to realize their full potential and instill confidence in their ability to make a positive impact on the organization. Consequently, it is suggested that followers guided by such leaders experience psychological empowerment [26]. Therefore, building on the previous discussion, we developed the following hypothesis:

  H2: Transformational leadership and psychological empowerment are positively correlated.

#### 1.1.3. Psychological Empowerment and OCBs

In accordance with the principles of social exchange theory, individuals operating within environments that foster empowerment are inclined to exhibit OCBs [27]. Numerous studies have identified psychological empowerment as a pivotal pathway leading to the manifestation of OCBs [28, 29]. These studies have found that when individuals perceive themselves as empowered within their organization, they are more likely to engage in behaviors that go beyond their formal job requirements and contribute to the overall well-being of the organization. Furthermore, Singh and Singh [30] conducted a study that revealed organizations fostering a sense of empowerment among their staff members experience positive outcomes, which are manifested in the form of OCBs. This suggests that psychological empowerment plays a crucial role in cultivating an organizational climate that encourages and motivates employees to engage in discretionary behaviors that benefit the organization and its members. Employees' OCBs are positively impacted by psychological empowerment because it creates a favorable work environment that improves their performance outside of their roles [8].

In conclusion, organizations that implement psychological empowerment programs are well-positioned to enhance OCBs and reduce intentions of employee turnover and retention [31]. Empowerment serves as a catalyst for motivating employees to exert their optimal efforts and signifies a heightened organizational commitment to goal achievement [32]. Building on the insights obtained from the aforementioned literature, we propose the following hypothesis:

  H3: Psychological empowerment has a positive and significant impact on OCBs among nurses.

#### 1.1.4. Psychological Empowerment as a Mediating Role

Earlier research findings have suggested that the skillful utilization of transformational leadership behaviors, such as idealized influence, intellectual stimulation, and individualized consideration, during organizational tasks can stimulate a sense of psychological empowerment among followers. This, in turn, has the potential to contribute to a higher level of organizational commitment [33, 34]. Empirical studies align with the principles of leadership theory. For instance, transformational leadership theory posits that the manner in which leaders and followers interact during the management of organizational activities can motivate followers to prioritize the interests of the organization over their own [35].

Moreover, in accordance with the psychological empowerment model, intrinsic task motivation has the potential to enhance employees' engagement and satisfaction with their work, irrespective of external rewards. The psychological empowerment model suggests that when employees feel a sense of autonomy, competence, meaningfulness, and impact in their work, they experience intrinsic motivation, which arises from the inherent satisfaction and enjoyment derived from the work itself. This intrinsic motivation is driven by the individual's internal desire to engage in the task and is not contingent on external rewards or incentives [36]. In line with this perspective, we propose that psychological empowerment, which has been found to have a positive correlation with OCBs, is closely linked to transformational leadership.

Transformational leaders, by exhibiting behaviors that inspire and empower their followers, create an environment conducive to psychological empowerment [26]. Furthermore, as job satisfaction arises from the fit between employees' jobs and their job values, fewer satisfied employees tend to perceive their jobs less matched with them and obtain less support (both extrinsic and intrinsic) when performing jobs [37].

Based on these insights, we propose the following mediating hypothesis:

  H4: Psychological empowerment mediates the relationship between transformational leadership and OCBs among nurses.

**Figure 1:**
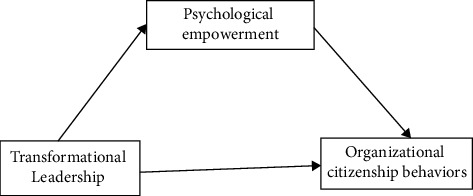
Conceptual framework developed for this study.

Based on the previous literature review and hypothesis development, the conceptual framework of the present study was developed as shown in Figure 1.

## 2. Materials and Methods

### 2.1. Study Design and Participants

This cross-sectional study was conducted at King Khalid Hospital in El-kharij Governorate. This hospital is equipped with high-quality health and medical departments that deliver efficient services to patients across various specialties. These departments include medical, surgical, operating room, radiology, dental, and intensive care units. Additionally, the hospital offers a dedicated section for psychological consultations, staffed by top doctors and psychologists. Furthermore, the hospital has a specialized department for medical endoscopy. The study utilized a convenience sampling method to recruit registered nurses from different age groups and departments within the hospital. The inclusion criteria required participants to be actively engaged in providing direct patient care on a full-time basis. To maintain consistency in the participant group, nursing interns and managers were excluded from the study. Out of the initial 357 nurses approached to participate in the study, a total of 305 nurses completed the self-reported questionnaires, resulting in a response rate of 85.4%.

### 2.2. Sample Size

The sample size was computed using the formula *n*=(*Z*^2^*∗σ*^2^)/*d*^2^, where “*n*” represents the required sample size, “*Z*” is the standardized normal deviation corresponding to a 95% confidence level and *α* = 0.05 (*Z* = 1.96 for a two-tailed test), “*σ*” denotes the expected standard deviation in the population (*σ* = 0.4) based on the study by Taghinezhad et al. [38], and “*d*” signifies the acceptable margin of error for the mean (*d* = 0.05). This calculation initially yielded a minimum sample size of 246, with provision for an anticipated 15% attrition rate. Subsequently, the final sample size was adjusted to 283 nurses.

### 2.3. Instrumentations

Data for the study were collected using three scales, all of which utilized a five-point Likert scale ranging from “1” representing “strongly disagree” to “5” representing “strongly agree.”

#### 2.3.1. Global Transformational Leadership Scale

The researchers employed the Global Transformational Leadership Scale developed by Carless in 2000 [38], to measure nurses' perceptions of transformational leadership demonstrated by their immediate leaders or managers. This scale contains seven items that assess four dimensions: idealistic impact, inspirational motivation, individual consideration, and intellectual stimulation and critical thinking. The reliability of the scale in this study was evaluated using Cronbach's alpha, which yielded a value of 0.74, indicating acceptable internal consistency.

#### 2.3.2. Psychological Empowerment Scale

The researchers utilized the Psychological Empowerment Scale developed by Spreitzer [23] to measure the various dimensions of psychological empowerment. This scale consists of twelve items, equally distributed across four dimensions: competence, meaning, self-determination, and impact. The internal consistency of the scale was assessed using Cronbach's alpha, which yielded a value of 0.89 for the total items, indicating high reliability. Furthermore, the subscales of the psychological empowerment scale demonstrated good internal consistency, with Cronbach's alpha values ranging from 0.71 to 0.90.

#### 2.3.3. OCBs Scale

The OCBs scale utilized in this study was developed by Podsakoff and his colleagues [39]. It consists of 12 items that are categorized into three dimensions: helping (6 items), civic virtue (3 items), and compliance (3 items). The internal consistency of the scale was assessed using Cronbach's alpha, which yielded a value of 0.70 for the total items. The subscales of the OCBs scale demonstrated satisfactory internal consistency, with Cronbach's alpha values ranging from 0.71 to 0.75. The utilized English scales demonstrated good reliability and validity in previous studies [40, 41].

The researchers opted to employ the English versions of the measurement scales in light of the fact that the registered nurses participating in the study routinely use English in their clinical practice and research was being conducted in English. Utilizing scales in English helped ensure the validity and reliability of responses by nurses sufficiently proficient in the language both professionally and academically. This approach reinforced the integrity of data collection and subsequent analysis within the study's methodological framework.

### 2.4. Data Collection

The data collection for this study occurred between March 2023 and August 2023, utilizing self-administered handwritten questionnaires. Participants were recruited through in-person meetings, during which they were provided with comprehensive information about the study, including its objectives, procedures, and their right to withdraw without facing any repercussions. Informed written consent was obtained from all participants before they proceeded to complete the questionnaire. To ensure confidentiality, participants were instructed to submit the completed questionnaires anonymously. Prior to the full implementation of the data collection, a pilot study was conducted with a sample of 29 registered nurses. The purpose of this pilot study was to identify any areas of the survey that required clarification or refinement. This iterative process allowed the researchers to enhance the clarity and user-friendliness of the survey items, thereby promoting the reliability of the data collection process. The estimated time for participants to complete the questionnaire ranged from 15 to 20 minutes. To ensure the accuracy and integrity of the collected data, the researchers conducted a thorough review of each received questionnaire. This rigorous review process was promptly undertaken to address any potential data omissions, inconsistencies, or missing information. By following these meticulous procedures, the researchers aimed to maintain the quality and reliability of the collected data.

The researchers carefully distributed the questionnaires to registered nurses, ensuring coverage across different shifts and working hours. By doing so, they aimed to capture a diverse range of perspectives. To discourage participants from providing hasty and careless responses, the researchers made a deliberate effort to collect the completed questionnaires at the end of the nurses' shifts. This approach allowed participants the autonomy to choose a suitable moment during their working hours to fill out the survey, promoting a conducive environment for thoughtful and thorough responses. The researchers believed that by providing participants with sufficient time and attention to complete the questionnaire, the quality and reliability of the collected data would be enhanced.

### 2.5. Ethical Considerations

This study obtained ethical approval from the institutional review board at Prince Sattam bin Abdulaziz University, identified by reference number SCBR-042-2023. Participation in the study was voluntary, and informed consent was secured with a guarantee of anonymity. Staff nurses were reassured that their responses would not impact performance evaluations, work status, or salaries. Completed questionnaires were submitted anonymously, and the data were treated confidentially, exclusively for research purposes.

### 2.6. Statistical Analysis

Data were meticulously organized, categorized, and presented in tables. Descriptive statistics, comprising numbers and percentages, were employed to delineate participants' demographic characteristics. The independent sample *t*-test and ANOVA test were utilized to compare means across diverse categories pertaining to personal characteristics and various study variables. The reliability of study variables was assessed using the Cronbach alpha test (*α*). Pearson correlation analysis explored the correlation matrix among different variables and subscales, with the strength of the correlation categorized according to Pearson's correlation coefficient. The process macro version 3.5 model 4 was employed for analyzing one independent variable (transformational leadership), one dependent variable (OCBs), and one mediator (psychological empowerment). The researchers utilized a biased bootstrap 95% confidence interval (CI) to assess the significance of total, direct, and indirect effects. The analysis was conducted using IBM SPSS Statistics 23, with the significance level set at *P* < 0.001.

## 3. Results

As shown in Table 1, most of the nursing staff were female (69.2%), less than half of them aged between 31 and 40 years, more than half were married (54.8%), two thirds had a bachelor's degree (66.2%), approximately two thirds of them have more than ten years of experience in nursing, while less than half of them usually worked in the morning shift (44.9%). 25.6% of the nursing staff worked in medical units. Finally, there were no statistically significant differences among transformational leadership, OCBs, and psychological empowerment with personal characteristics.

Table 2 demonstrates nurses who recorded mean scores of 3.38, 3.40, and 3.54 for transformational leadership, psychological empowerment, and OCBs, respectively. Among the dimensions of psychological empowerment, the highest mean was observed in the meaning dimension (3.53 ± 0.83). Similarly, the compliance dimension recorded the highest mean for OCBs (3.69 ± 0.60).

The findings in Table 3 reveal a positive correlation between staff nurse's transformational leadership and psychological empowerment (*r* = 0.507, *P* < 0.001), as well as with OCBs (*r* = 0.445, *P* < 0.001). Additionally, a positive correlation was observed between psychological empowerment and OCBs (*r* = 0.451, *P* < 0.001).

Table 4 further illustrates that transformational leadership has a positive impact on psychological empowerment (*B* = 0.433, *p* < 0.001) and OCBs (*B* = 0.208, *p* < 0.001) among the studied nurses. Furthermore, psychological empowerment positively influences OCBs among the participants (*B* = 0.254, *p* < 0.001). Notably, psychological empowerment was identified as a partial mediator in the relationship between transformational leadership and OCBs among the studied nurses (*B* = 0.110, CI: 0.058–0.166).

## 4. Discussion

The present research study aimed to investigate the relationship between transformational leadership and OCBs in the nursing context, with psychological empowerment acting as a mediating factor.

Our findings demonstrated a positive impact of transformational leadership on OCBs among nursing staff, supported H1. This is attributable to the development of a supportive work environment characterized by open communication, collaboration, and mutual respect. In such environments, employees are more likely to engage in OCBs, as they experience a stronger sense of belonging and commitment to the organization's goals. This, in turn, leads to improved job satisfaction and overall organizational performance [7, 16, 32].

Our study aligns with the findings of Qiu et al. [42], who conducted research across eight hospitals in China and revealed that a leadership style characterized by role modeling, integrity, and ethics had a direct impact on OCBs among nursing staff. Similarly, a study conducted by Aloustani et al. [43] among nurses from twelve teaching hospitals in Tehran demonstrated that a leadership style that promotes an ethical climate has the ability to enhance OCBs. This indicates that when nurses perceive their leaders as ethical and morally upright, they are more likely to exhibit behaviors that benefit the organization as a whole.

Furthermore, the study by López-Ibort et al. [44] in Spain, which included nurses and supervisors from nine public hospitals in the autonomous community of Aragon, revealed that a leadership style that fosters quality nurse-supervisor relationships encourages OCBs among participants. Rimatanti et al. [45] also found a positive and significant effect of transformational leadership on OCBs among a purposive sample of nurses in Indonesia. Similarly, Hall [46] conducted a study among registered nurses in the United States and found that leadership skills play a crucial role in promoting OCBs. Moreover, transformational leadership in nursing unit managers improves nurses' empowerment, performance, job satisfaction, and organizational commitment [47]. However, it is important to note that the study by Kim [48] in South Korea found no significant influence of transformational leadership on OCBs among employees of the Gwangju Metropolitan City government. This discrepancy may be attributed to cultural or contextual differences between the nursing context and the government setting.

The study findings revealed that transformational leadership had a significant and positive relationship with psychological empowerment, supporting H2. This implies that transformational leaders foster supportive and inclusive environments that prioritize the growth and development of employees. In such environments, individuals feel valued, acknowledged, and supported in their personal and professional journeys. These elements significantly contribute to the enhancement of employees' sense of competence, autonomy, and self-determination, which are fundamental aspects of psychological empowerment [49]. Moreover, transformational leaders actively engage in coaching and mentoring behaviors, offering valuable guidance and feedback that aid in the skill-building and confidence-boosting processes of employees. This, in turn, further strengthens their overall sense of empowerment [50].

These findings are consistent with the meta-analysis study conducted by Schermuly et al. [22], which investigated the effects of various leadership styles on psychological empowerment. Their study found that transformational leadership contributes to psychological empowerment. Additionally, the Chinese study by Hua et al. [51] revealed that nurse managers who adopt a transformational leadership style enhance psychological empowerment among nurses. The mediation study of Zhang et al. [52] found that transformational leadership had a positive and significant impact on psychological empowerment.

The findings of the present study showed that psychological empowerment has a positive and significant impact on OCBs among nurses, supporting H3. This may be due to psychological empowerment is associated with higher levels of job satisfaction and organizational commitment among nurses, which further encourages them to exhibit OCBs as a form of positive citizenship behavior that enhances the overall effectiveness and success of the organization [53, 54]. Our findings are supported by the study of Turnipseed and VandeWaa [55] that was carried out among nurses in a medium-size urban general hospital and revealed that there are differential relationships between the dimensions of psychological empowerment and the dimensions of OCBs. Also, the meditation study of AlHammadi and Elanain [12] revealed that psychological empowerment had positive and direct impact on OCBs. Moreover, Jafari et al. [29] found that psychological empowerment associated positively with positive organizational behavior among nurses working in university hospitals affiliated to the Kermanshah University of Medical Sciences in Iran.

Our findings revealed that psychological empowerment mediates the relationship between transformational leadership and OCBs among nurses, supporting H4 and indicating that transformational leaders may foster a work environment where nurses feel empowered to contribute positively to the organization, leading to increased OCBs. These findings in the same line with the study of Saira et al. [8] revealed that transformational leadership positively impacts employee outcomes like OCBs and turnover intention through increased psychological empowerment. Similarly, in northeastern United States, Dust et al. [56] found that psychological empowerment mediated relationships between transformational leadership and employee task performance and OCBs. Moreover, Cheng et al. [25] showed that transformational leadership shows positive and negative effects on deep acting and surface acting, respectively. The positive effect on deep acting is partially mediated by psychological empowerment, while the negative effect on surface acting is fully mediated by psychological empowerment. The study of Shapira-Lishchinsky and Benoliel [57] found that nurses' psychological empowerment and their head nurses' authentic leadership positively impact their OCBs, tardiness, absenteeism, and intent to leave the hospital. The study of Han et al. [58] indicates a significant direct effect of transformational leadership on psychological empowerment, organizational commitment, and OCBs. Moreover, transformational leadership also shows an indirect effect on employees' OCBs, which, in turn, is identified as the primary factor that influences knowledge sharing among employees of five large companies in South Korea. Huang et al. [59] found that psychological empowerment positively mediated the relationships between both transformational and contingent reward leaderships and organizational commitment among university members in China.

Moreover, psychological empowerment plays a crucial role in the relationship between transformational leadership and positive consequences in nursing context. For instance, the investigation of Masood and Afsar [60] found that transformational leadership fosters innovative work behavior among nursing staff by increasing psychological empowerment, intrinsic motivation, and knowledge sharing behavior. Also, the study of Zhang et al. [52] found that transformational leadership and psychological empowerment significantly improve nurses' innovative behavior during the COVID-19 pandemic.

## 5. Conclusion

In conclusion, his study emphasizes the role of psychological empowerment as a mediating factor in the relationship between transformational leadership and OCBs among nurses. It underscores how leadership styles shape organizational culture and employee behaviors. Organizations that prioritize and nurture transformational leadership are positioned to witness increased levels of voluntary, positive behaviors, which ultimately enhance the overall success and well-being of the organization.

### 5.1. Limitations of the Study

This study is limited in its scope to a specific setting and a distinct group of nurses, which may restrict the generalizability of the findings to other healthcare contexts, nursing professions, or diverse healthcare professionals. Therefore, future research should aim to replicate the current study in diverse healthcare settings and among different healthcare professionals to assess the generalizability of the observed relationships. The utilization of a cross-sectional design captures only a snapshot of the relationships at a specific moment, cautioning against drawing causal conclusions. To gain more insight into the causal dynamics over time, longitudinal studies could be employed. Additionally, supplementing self-reported data with objective performance measures or supervisor ratings would enhance the validity of the study. The reliance on self-reported data through questionnaires introduces the potential for response bias, as participants may provide socially desirable answers or not fully reflect their actual behaviors. To address this limitation, incorporating a mixed-methods approach that includes qualitative insights could provide a more comprehensive understanding of the subjective experiences of nurses and leaders. Lastly, conducting intervention studies focused on leadership training programs may offer practical insights into enhancing transformational leadership behaviors and their subsequent impact on psychological empowerment and OCBs.

### 5.2. Implications of the Study

Nurse managers should cultivate and strengthen their transformational leadership behaviors to empower nurses psychologically, thereby fostering a positive workplace culture and promoting OCBs. To achieve this, the development of training programs focused on enhancing psychological empowerment among nurses is recommended. These programs should emphasize the significance of nurses' roles, provide opportunities for skill development, and promote autonomy.

## Figures and Tables

**Table 1:** Personal characteristics of the studied nurses and differences in the study variables.

Characteristics	*N*	%	Transformational leadership	Psychological empowerment	Organizational citizenship behavior
			Mean ± SD	Mean ± SD	Mean ± SD
*Age (years)*					
20–30	73	23.9	3.25 ± 0.75	3.30 ± 0.64	3.48 ± 0.53
31–40	134	43.9	3.45 ± 0.74	3.50 ± 0.65	3.58 ± 0.54
41–50	85	27.9	3.35 ± 0.75	3.34 ± 0.61	3.50 ± 0.54
<50	13	4.3	3.45 ± 0.43	3.41 ± 0.53	3.64 ± 0.37
*f*/*p*			1.31/0.27	1.92/0.13	0.79/0.50

*Gender*					
Male	94	30.8	3.29 ± 0.71	3.45 ± 0.60	3.52 ± 0.55
Female	211	69.2	3.41 ± 0.75	3.39 ± 0.65	3.54 ± 0.53
*t*/*p*			1.08/0.16	0.75/0.46	0.35/0.73

*Marital status*					
Single	112	36.7	3.47 ± 0.58	3.43 ± 0.55	3.49 ± 0.4
Married	167	54.8	3.34 ± 0.82	3.39 ± 0.70	3.57 ± 0.61
Divorced	20	6.6	3.15 ± 0.81	3.48 ± 0.57	3.53 ± 0.56
Widowed	6	2.0	3.28 ± 0.65	2.99 ± 0.28	3.40 ± 0.31
*f*/*p*			1.43/0.23	1.04/0.37	0.56/0.64

*Educational level*					
Technical education	62	20.3	3.28 ± 0.65	3.41 ± 0.66	3.57 ± 0.43
Bachelor's degree	202	66.2	3.38 ± 0.79	3.39 ± 0.65	3.51 ± 0.58
Postgraduate degree	41	13.4	3.51 ± 0.61	3.46 ± 0.53	3.60 ± 0.41
*f*/*p*			1.14/0.32	0.20/0.82	0.62/0.54

*Years of experience*					
<5	45	14.8	3.19 ± 0.73	3.26 ± 0.69	3.42 ± 0.53
6–10	78	25.6	3.48 ± 0.79	3.47 ± 0.68	3.57 ± 0.59
10–15	93	30.5	3.40 ± 0.74	3.49 ± 0.62	3.53 ± 0.51
>15	89	29.2	3.37 ± 0.71	3.34 ± 0.57	3.56 ± 0.51
*f*/*p*			1.49/0.22	1.94/0.12	0.88/0.45

*Shift*					
Morning	137	44.9	3.35 ± 0.81	3.41 ± 0.68	3.53 ± 0.58
Afternoon	53	17.4	3.35 ± 0.71	3.38 ± 0.56	3.54 ± 0.42
Night	48	15.7	3.46 ± 0.74	3.49 ± 0.63	3.55 ± 0.56
Morning and evening	67	22.0	3.38 ± 0.64	3.34 ± 0.61	3.53 ± 0.49
*f*/*p*			0.29/0.84	0.58/0.63	0.01/0.99

*Working unit*					
Emergency	45	14.8	3.35 ± 0.69	3.37 ± 0.60	3.48 ± 0.48
Medical	78	25.6	3.34 ± 0.84	3.42 ± 0.67	3.51 ± 0.56
Surgical	64	21.0	3.41 ± 0.78	3.45 ± 0.68	3.51 ± 0.55
Dialysis	38	12.5	3.39 ± 0.75	3.41 ± 0.72	3.51 ± 0.59
Outpatient	42	13.8	3.36 ± 0.56	3.47 ± 0.48	3.59 ± 0.56
ICU	26	8.5	3.35 ± 0.73	3.17 ± 0.63	3.65 ± 0.38
Operative	12	3.9	3.48 ± 0.69	3.39 ± 0.51	3.69 ± 0.53
*f*/*p*			0.11/0.99	0.78/0.59	0.58/0.75

^*∗*^Significant (*P* < 0.05). (*F*) ANOVA test. (*t*) *t*-test.

**Table 2:** Descriptive statistics of the study variables.

The study variables	Min-max	Mean ± SD
Transformational leadership	1.14–4.86	3.38 ± 0.74
Psychological empowerment	1.42–4.67	3.40 ± 0.64
Meaning	1.00–5.00	3.53 ± 0.83
Competence	1.00–5.00	3.37 ± 0.84
Self-determination	1.00–5.00	3.40 ± 0.87
Impact	1.00–5.00	3.32 ± 0.84
OCBs	1.67–4.92	3.54 ± 0.53
Helping	1.17–4.83	3.52 ± 0.63
Civic virtue	1.00–5.00	3.41 ± 0.80
Compliance	2.00–5.00	3.69 ± 0.60

OCBs: organizational citizenship behaviors.

**Table 3:** Correlation among the study variables.

The study variables	1	2	3	4	5	6	7	8	9	10
(1) Transformational leadership	1.00									
(2) Psychological empowerment	0.507^*∗∗∗*^	1.00								
(3) Meaning	0.298^*∗∗∗*^	0.697^*∗∗∗*^	1.00							
(4) Competence	0.424^*∗∗∗*^	0.784^*∗∗∗*^	0.372^*∗∗∗*^	1.00						
(5) Self-determination	0.464^*∗∗∗*^	0.782^*∗∗∗*^	0.367^*∗∗∗*^	0.531^*∗∗∗*^	1.00					
(6) Impact	0.340^*∗∗∗*^	0.756^*∗∗∗*^	0.376^*∗∗∗*^	0.468^*∗∗∗*^	0.444^*∗∗∗*^	1.00				
(7) OCBs	0.445^*∗∗∗*^	0.451^*∗∗∗*^	0.261^*∗∗∗*^	0.362^*∗∗∗*^	0.445^*∗∗∗*^	0.289^*∗∗∗*^	1.00			
(8) Helping	0.366^*∗∗∗*^	0.394^*∗∗∗*^	0.217^*∗∗∗*^	0.326^*∗∗∗*^	0.397^*∗∗∗*^	0.244^*∗∗∗*^	0.863^*∗∗∗*^	1.00		
(9) Civic virtue	0.385^*∗∗∗*^	0.364^*∗∗∗*^	0.226^*∗∗∗*^	0.290^*∗∗∗*^	0.339^*∗∗∗*^	0.241^*∗∗∗*^	0.725^*∗∗∗*^	0.349^*∗∗∗*^	1.00	
(10) Compliance	0.294^*∗∗∗*^	0.285^*∗∗∗*^	0.167^*∗∗∗*^	0.212^*∗∗∗*^	0.289^*∗∗∗*^	0.191^*∗∗∗*^	0.764^*∗∗∗*^	0.495^*∗∗∗*^	0.499^*∗∗∗*^	1.00

OCBs: organizational citizenship behaviors ^*∗∗∗*^*p* < 0.001.

**Table 4:** Mediating effect of psychological empowerment between transformational leadership and organizational citizenship behavior.

	*B*	SE	*t*	LL CI	UL CI
Transformational leadership to psychological empowerment	0.433	0.042	10.227^*∗∗∗*^	0.350	0.517
Transformational leadership to OCBs	0.208	0.041	5.095^*∗∗∗*^	0.128	0.289
Psychological empowerment to OCBs	0.254	0.048	5.307^*∗∗∗*^	0.160	0.348
Total effect	0.319	0.037	8.648^*∗∗∗*^	0.246	0.391
Direct	0.209	0.041	5.095^*∗∗∗*^	0.128	0.289
Indirect	0.110	0.028		0.058	0.166

OCBs: organizational citizenship behaviors/^*∗∗∗*^*p* < 0.001.

## Data Availability

The data that support the findings of this study are available on reasonable request from the corresponding author.
